# Treating exertional heat stroke: Limited understanding of the female response to cold water immersion

**DOI:** 10.3389/fphys.2022.1055810

**Published:** 2022-11-25

**Authors:** Kate P. Hutchins, Geoffrey M. Minett, Ian B. Stewart

**Affiliations:** School of Exercise and Nutrition Sciences, Queensland University of Technology, Kelvin Grove, QLD, Australia

**Keywords:** cold water immersion, exertional heat stroke, female, core temperature, hyperthermia

## Abstract

According to an expansive body of research and best practice statements, whole-body cold water immersion is the gold standard treatment for exertional heat stroke. However, as this founding evidence was predominantly drawn from males, the current guidelines for treatment are being applied to women without validation. Given the recognised differences in thermal responses experienced by men and women, all-encompassing exertional heat stroke treatment advice may not effectively protect both sexes. In fact, recent evidence suggests that hyperthermic women cool faster than hyperthermic men during cold water immersion. This raises the question of whether overcooling is risked if the present guidelines are followed. The current mini-review examined the literature on women’s response to cold water immersion as a treatment for exertional heat stroke and aimed to clarify whether the current guidelines have appropriately considered research investigating women. The potential implications of applying these guidelines to women were also discussed.

## Introduction

Exertional heat stroke (EHS) is defined by an elevation in core temperature above 40°C together with impaired central nervous system function (e.g., irritability, confusion, altered consciousness) (Bouchama et al., 2022). These hallmarks reflect an overwhelmed thermoregulatory system whereby the metabolic heat load owing to muscle activity exceeds the heat load that can be emitted from the body *via* heat loss pathways. Hence, this form of heat stroke most often presents in healthy, active populations during strenuous or prolonged physical exertion, especially in thermally challenging environments (e.g., high temperature, high humidity, solar radiation, lack of wind flow, and insulative clothing). In these circumstances, core temperature values above 40°C have been documented in well-trained male and female athletes (Racinais et al., 2019) and male defence personnel (Byrne et al., 2006) without symptoms of central nervous system dysfunction or the presence of exertional heat stroke. Thus, a core temperature above 40°C alone is not sufficient to diagnose EHS.

Timing is critical in the treatment of EHS. Identification and diagnosis should be rapid and initiation of treatment immediately as the patient’s risk of severe tissue damage, multi-organ failure, and death is directly related to the duration and magnitude of core temperature elevation (Casa et al., 2007). Reducing core temperature below the threshold for critical cell damage (∼42°C) within 30 min of diagnosis, termed ‘the golden half-hour’, is the central aim of EHS treatment (Casa et al., 2012). This treatment guidance is outlined in sport and occupational position or consensus statements and best practice pre-hospital standards (American College of Sports Medicine, National Athletic Trainers’ Association, International Olympic Committee—Adams et al., 2021; Belval et al., 2018; Casa et al., 2015; Hosokawa et al., 2021; Roberts et al., 2021). The guidelines aim to provide those administering care with time-critical milestones that, when closely followed, will optimise the health outcomes of EHS cases.

The gold standard and recommended cooling treatment for EHS is cold water immersion (CWI) (Casa et al., 2007). The treatment involves immersion of the whole-body in cold water until a safe core temperature is restored. While simplistic, CWI is a highly effective cooling method, reducing core temperature quicker than all other interventions in controlled studies and field interventions (Douma et al., 2020). An expanse of high-quality research backs this method (see systematic reviews and meta-analyses, Douma et al., 2020; Zhang et al., 2015), while critical assessments of previous guidelines (Brearley, 2019; Hosokawa et al., 2019; Laitano et al., 2019) have also been instrumental in positioning CWI as the premier EHS treatment. This body of research informs the existing recommendations, with the following parameters identified as being critical to EHS treatment success: 1) immediate identification and initiation of treatment, 2) immersion in cold water <10°C, 3) water to be circulating, 4) immersion up to the clavicle, 5) rectal temperature, measured *via* thermistor, to a minimum safety cooling limit of 38.6°C, or 6) without a rectal temperature measure only immerse for 10–15 min (Casa et al., 2007, 2015; Roberts et al., 2021). Importantly, however, it is unclear whether women’s response to CWI has been considered in developing the current EHS guidelines.

In the broader topic of exercise thermoregulation, inadequate female data has been reported with less than 18% of research involving female participants in the 10 years preceding 2019 (Hutchins et al., 2021). Such disparity in the representation of women is also likely present in CWI research and is potentially limiting our understanding of how women respond to this treatment. Female under-representation in exercise science and sports medicine literature (Costello et al., 2014) has previously resulted in evidence-based strategies formed from research conducted in men being erroneously applied to women without validation or consideration for differences between the sexes (D’Lauro et al., 2022; Smith et al., 2022). In a sporting context, the implications may be that an athlete’s performance is not optimised, whereas in treating an individual with EHS the implications could be far more severe.

Previous CWI research indicates there may be a difference between the sexes in the most critical factor in EHS treatment, cooling rates (Lemire et al., 2009; Boehm and Miller, 2019). Specifically, hyperthermic women may cool more rapidly than hyperthermic men during CWI (Armstrong et al., 1996; Lemire et al., 2009). This divergent response is frequently attributed to individual variability in physical characteristics between the sexes. However, sex differences in metabolic sensitivity, post-exercise muscle temperature, plasma volume, and female reproductive hormone (synthetic and natural) may also be responsible.

While rapid cooling is favourable in treating EHS, the potential risk of hypothermia exists if core temperature is not closely monitored *via* an in-dwelling rectal thermometer. Unfortunately, such temperature monitoring equipment is seldom used or accessible in the field despite its importance in EHS diagnosis and treatment (Mazerolle et al., 2010; Hirschhorn et al., 2021). Thus it begs the question, are the current EHS guidelines optimised to protect women?

These observations have highlighted the need to review the research on women’s response to CWI as a treatment for EHS, clarify whether women have been included in the research underpinning the guidelines and discuss the potential implications of applying these guidelines to women.

## Cold water immersion in hyperthermic women

Although the absolute number of EHS incidences in women is less than men, women are not exempt from this life-threatening condition (Belval et al., 2020; DeMartini et al., 2015; Giersch et al., 2022). As women’s representation in sport, the military and industry continues to grow under the current societal and legislative climate (Ministry of Defence, 2014; Brushlinksy et al., 2017; International Olympic Committee, 2018) so too will the number of females exposed to exertional and environmental heat loads and therefore at risk of EHS.

The influence of female body composition, morphology, and hormonal milieu on thermoregulation has been the focus of recent opinion pieces (Notley et al., 2021) and reviews (Baker et al., 2020; Giersch et al., 2020). However, few studies investigating CWI as an EHS treatment have involved female participants (Table 1; Clements et al., 2002; Proulx et al., 2003; 2006; Gagnon et al., 2010; Hostler et al., 2014; Butts et al., 2016; Nye et al., 2017). Even fewer publications have explicitly examined the response of hyperthermic women to whole-body CWI (Armstrong et al., 1996; Lemire et al., 2009; DeMartini et al., 2015; Koenig et al., 2022). Small samples of women and the aggregation of female and male data in previous work have limited our understanding of CWI in women (Table 1; Boehm and Miller, 2019; Douma et al., 2020). In a recent review of cooling techniques for EHS, no whole-body CWI studies exclusively involved women, and in four of the six studies involving women, male and female data were aggregated (Douma et al., 2020). Based on these data aggregation practices, it may be possible that the authors of these former works (including recent publications) did not believe individual responses to CWI differed between the sexes given the evidence investigating a sex difference was limited. If this was the case, their choice to data aggregate was justifiable and contributed to the statistical power of the outcomes.

**TABLE 1:** Study design and results of cold water immersion research involving hyperthermic women or females with exertional heat stroke.

Laboratory Research									
Citation	*n* = (M/F)	Age ± SD (yrs)	Study design	Type of heatstroke	Description of cooling Method(s)	Pre-immersion Trec ± SD (°C)	Cooling time ± SD (min)	Final Trec ± SD (°C)	Cooling rate ± SD (°C∙min^−1^)
		Mass ± SD (kg)							
		Body fat ±SD (%)							
Butts (2016)	*n* = 14 (8/6)*	26 ± 5	CWI: euhydration (EU)	Exertional hyperthermia (volitional intensity, lab)	CWI: 2°C from iliac crest to xiphoid process	EU: 39.3 ± .3	EU: 9.6 ± 3.6	EU: 38.1 ± 0.2	EU: 0.14 ± 0.05
		72 ± 9	CWI: hypohydration (HY)			HY: 39.3 ± .4	HY: 11.8 ± 3.4	HY: 38.2 ± 0.3	HY: 0.11 ± 0.05
		21 ± 7							
Clements (2002)	*n* = 17 (14/3)*	28 ± 2	passive air cooling	Exertional hyperthermia (fixed intensity, field)	Passive: air temp 29°C CWI: 5/14°C from shoulders to hip joint	Passive: 39.3 ± 0.2	fixed at 12 min	Passive: 37.8 ± 0.2	Passive: 0.10 ± 0.01
		69 ± 2	CWI: 5°C			CWI (5°C): 39.5 ± 0.2		CWI (5°C): 37.2 ± 0.1	CWI (5°C): 0.16 ± 0.01
		11 ± 1	CWI: 14°C			CWI (14°C): 39.6 ± 0.2		CWI (14°C): 37.5 ± 0.1	CWI (14°C): 0.16 ± 0.01
Gagnon (2010)	*n* = 10 (6/4)*	22 ± 3	CWI: cooling limit T_rec_ 37.5°C	Exertional hyperthermia (fixed intensity, lab)	CWI: 2°C immersed in recumbent position in circulated water	CWI (37.5°C): 39.7 ± 0.1	CWI (37.5°C): 17 ± 6	CWI (37.5°C): 37.6 ± 0.0	CWI (37.5°C): 0.14 ± 0.05
		68 ± 11	CWI: cooling limit T_rec_ 38.6°C			CWI (38.6°C): 39.6 ± 0.1	CWI (38.6°C): 9 ± 3	CWI (38.6°C): 38.6 ± 0.1	CWI (38.6°C): 0.12 ± 0.04
		17 ± 5							
Koenig (2022)	*n* = 16 (16/16)	22 ± 2	CWI: high body surface area-to-lean mass ratio (High) CWI: low body surface area-to-lean mass ratio (Low)	Exertional hyperthermia (fixed intensity, lab)	CWI: 10°C circulated water to the neck	∼39.5^1^	High: ∼6^2^Low: ∼6^2^	∼38.0^1^	High: 0.27 ± 0.07 Low: 0.26 ± 0.09
		65 ± 9							
		30 ± 6							
Lemire (2009)	*n* = 19 (10/9)	F: 24 ± 3	CWI: females (F)	Exertional hyperthermia (fixed intensity, lab)	CWI: 2°C immersed in recumbent position in circulated water	F: 39.6 ± 0.1	F: 11 ± 5	∼37.5^1^	F: 0.22 ± 0.07
		64 ± 9							
		24 ± 6							
		M: 25 ± 4	CWI: males matched for body surface area-to-mass ratio (M)			M: 39.6 ± 0.1	M: 18 ± 5		M: 0.12 ± 0.03
		74 ± 9							
		14 ± 3							
Nye (2017)	*n* = 18 (9/9)*	24 ± 4	CWI	Exertional hyperthermia (fixed intensity, field)	CWI: 5–10°C to the mid-chest	CWI: 38.6 ± 0.4	CWI: ∼15^2^	∼37.5^1^	CWI: 0.07 ± 0.04
		59 ± 17	Ice sheets (IS)		IS: 5–10°C sheets covering major arteries	IS: 38.6 ± 0.5	IS: ∼22^2^		IS: 0.05 ± 0.05
		—	Polar Life Pod (PLP)		PLP: 5–10°C head, neck, torso partially immersed	PLP: 38.4 ± 0.7	PLP: ∼26^2^		PLP: 0.04 ± 0.08
Proulx (2006) Proulx (2003)	*n* = 7 (4/3)*	22 ± 2	CWI: 2°C	Exertional hyperthermia (fixed intensity, lab)	CWI: 2/8/14/20°C circulated water to the clavicle	CWI (2°C): 39.8 ± 0.3	CWI (2°C): 5.5	CWI (2°C): 37.5 ± 0.1	CWI (2°C): 0.35 ± .14
		68 ± 11	CWI: 8°C			CWI (8°C): 39.8 ± 0.2	CWI (8°C): 8.0	CWI (8°C): 37.5 ± 0.1	CWI (8°C): 0.19 ± .07
		17 ± 8	CWI: 14°C			CWI (14°C): 39.8 ± 0.3	CWI (14°C): 11.0	CWI (14°C): 37.5 ± 0.0	CWI (14°C): 0.15 ± 0.06
			CWI: 20°C + immersion to T_rec_ 37.5°C			CWI (20°C): 39.8 ± 0.2	CWI (20°C): 14.0	CWI (20°C): 37.5 ± 0.0	CWI (20°C): 0.19 ± 0.10

**Retrospective Field Research**									
Citation	*n* = (M/F)	Age ± SD (yrs)	Study design	Type of heatstroke	Description of cooling Method(s)	Pre-immersion Trec ± SD (°C)	Cooling time ± SD (min)	Final Trec ± SD (°C)	Cooling rate ± SD (°C∙min^−1^)
		Mass ± SD (kg)							
		Body fat ±SD (%)							
Armstrong (1996)	*n* = 14 (13/1)	F: 41	Retrospective field study mass participation event	Exertional heat stroke	CWI: 1–3°C torso and legs immersed	F: 41.9M: 41.6 ± 0.6	F: 11 M: 17 ± 8	F: 38.9 M: 38.6 ± 0.8	F: 0.27 M: 0.20 ± 0.07
		—							
		—							
		M: 34 ± 13							
		—							
		—							
DeMartini (2015)	*n* = 179 (110/69)	F: 34 ± 13	Retrospective field study mass participation event	Exertional heat stroke	CWI: 10°C whole-body or torso immersed with ice towels on limbs	F: 41.2 ± 0.7 M: 41.4 ± 0.7	F: 11 M: 10	∼38.8^1^	F: 0.22 ± 0.10 M: 022 ± 0.12
		—							
		—							
		M: 36 ± 12							
		—							
		—							
Hostler (2014)	*n* = 10 (6/4)*	29 ± 6	Retrospective field study mass participation event	Exertional heat stroke	CWI: temperature not specified, immersion of torso and thighs	CWI: 41.5 ± 0.8	CWI: 20 ± 5	CWI: 38.8 ± 0.9	CWI: 0.13 ± 0.04
		—							
		—							

CWI—cold water immersion; SD—standard deviation; T_re_–rectal temperature.

^*^ Male and female data aggregated.

^1^ Only core temperature cut off or safety cooling limit provided not analysed data.

^2^ Calculated from pre-immersion, post-immersion rectal temperature and cooling rate.

An early retrospective examination of medical tent data from the Falmouth Road Race by Armstrong and colleagues (1996) reported substantial inter-individual variability in the cooling rates of competitors diagnosed with EHS (range: 0.12–0.34°C∙min^−1^). One female case was recorded, with their cooling rate higher than the average of 13 males during immersion in ice water (1–3°C; woman 0.27°C∙min^−1^; men 0.20°C∙min^−1^) (Armstrong et al., 1996). However, little can be concluded from a single record. Over 60 incidences of EHS in female runners were examined in a more recent review of Falmouth medical records (DeMartini et al., 2015). This work reported no difference in the cooling rate of men and women (0.22°C∙min^−1^) during immersion in cold (10°C) water, although large variability in cooling rates were also highlighted (range 0.04–0.56°C∙min^−1^; DeMartini et al., 2015).

Unfortunately, no anthropometric or situational details were available from these investigations due to the nature of field records. As such, how individual cooling responses may be associated with distinctive physical characteristics or actions leading up to or during the event cannot be evaluated. More specifically, data of this nature and volume may have offered an insight into the cooling responses of a more heterogeneous population.

## Laboratory research

As body composition and morphology are key determinants of heat storage and exchange with the environment (Anderson, 1999), physical characteristics have been shown to alter the cooling rate during CWI (White et al., 1992). For example, factors such as adiposity (Baker and Daniels, 1956) and lean body mass percentage (Anderson et al., 1995) reduce heat loss in normothermic individuals during CWI, whereas cooling rate is greater in those with larger surface area-to-mass ratios (McArdle et al., 1984a). However, exercise-induced hyperthermic individuals do not reflect these same effects (Lemire et al., 2009). Differences in heat distribution and muscle and skin blood flow associated with exercise-induced hyperthermia have been shown to elevate the core-to-skin and skin-to-water thermal gradients, consequently minimising the insulative effect of skeletal muscle and promoting heat loss to the environment (Scott et al., 2004; Lemire et al., 2008; 2009).

In hyperthermic males, Lemire et al. (2008) reported that a 10% difference in body adiposity did not influence the core temperature cooling rate in 8°C water. In another study, where body surface area-to-mass ratio was matched between the sexes, Lemire et al. (2009) identified significantly quicker cooling rates in females than males (women 0.22°C∙min^−1^; men 0.12°C∙min^−1^). However, the correlation between body surface area-to-lean mass ratio and cooling rate outlined by Lemire et al. (2009) to explain this sex difference (r = 0.70, *p* = .001) appears to result from another confounding variable that remains unidentified.

**FIGURE 1:**
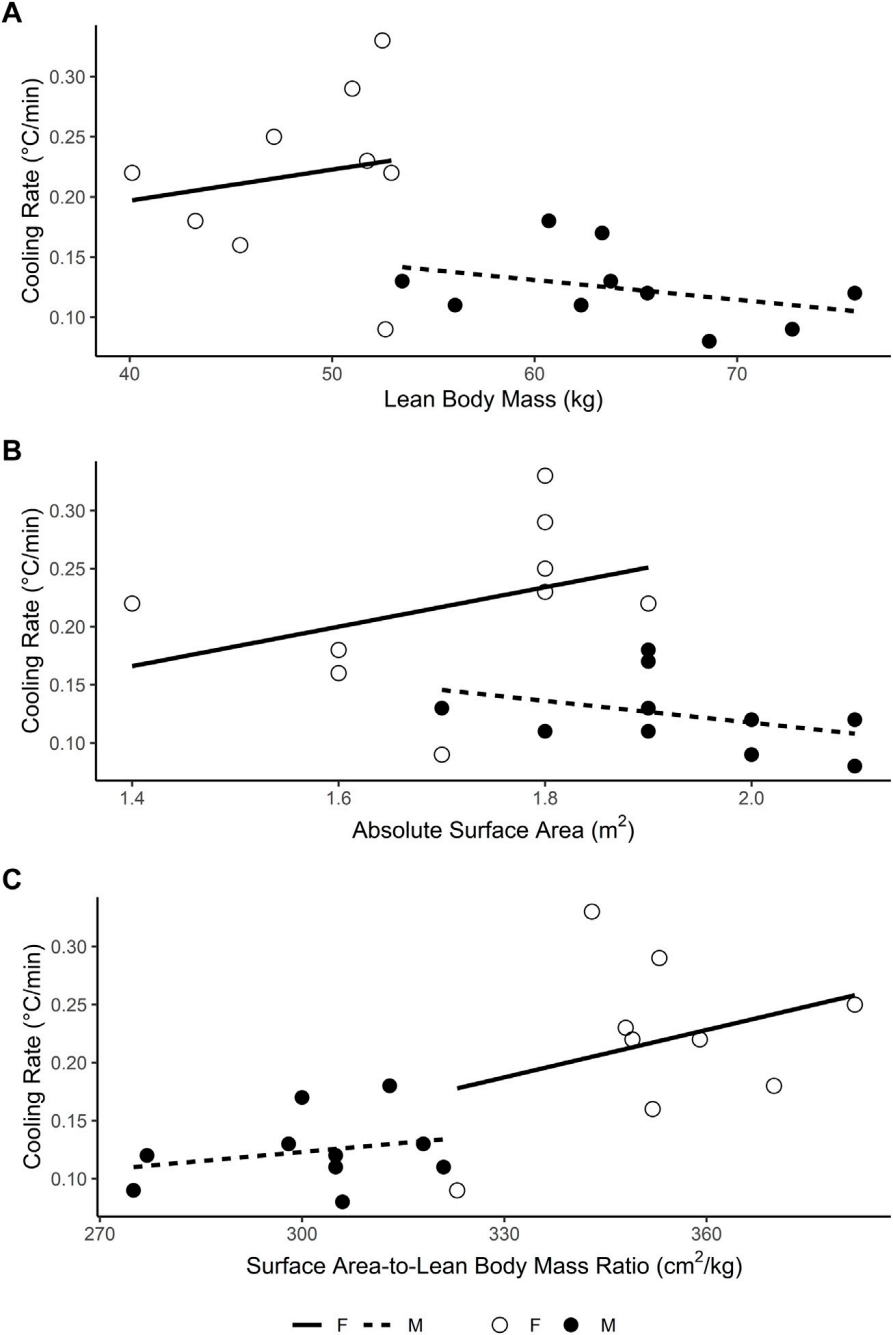
Relationship between **(A)** lean body mass **(B)** absolute surface area and **(C)** surface area-to-lean mass ratio and rectal temperature cooling rate in male (filled circles) and female (open circles) participants during cold water immersion (2°C) after exercise-induced hyperthermia. Data obtained with permission from Lemire et al. (2009).

Secondary analysis of the data (obtained from Lemire et al., 2009 with permission, Figure 1) shows that body surface area-to-lean mass ratio is not related to cooling rate independently for males (r = 0.26, bootstrapped 95% CI_BCa_ [−0.26, 0.77], *p* = .474; Figure 1C), nor females (r = 0.32, bootstrapped 95% CI_BCa_ [−0.67, 0.90] *p* = .403; Figure 1C). Lemire et al.’s (2009) conclusion that the faster cooling rates in females “may be attributed to physical differences in lean body mass”, appears to be an invalid interpretation of their data, as neither males (r = −0.36 [−0.72, 0.22], *p* = .307; Figure 1A), nor females (r = 0.17, [−0.55, 0.74], *p* = .656; Figure 1A) displayed any relationship between lean body mass and cooling rates. Strikingly, the point estimate of the correlations is in opposite directions (Figure 1A; analysis and code available in Supplementary Datasheet S1).

A retrospective analysis of six data sets (*n* = 62 individuals, 14 females) (Proulx et al., 2006; Lemire et al., 2009; Gagnon et al., 2010; Friesen et al., 2014; Flouris et al., 2014; 2015) by Poirier et al. (2018) found similarly weak, though statistically significant, correlations between cooling rate and height (r = 0.22, *p* = .030), body mass (r = 0.30, *p* = .005), lean body mass (r = 0.37, *p* < .001), body surface area-to-lean body mass ratio (r = −0.35, *p* < .001) and body surface area-to-body mass ratio (r = −0.28, *p* = .008) of the heterogeneous group. Subsequent stepwise multiple regression was undertaken, and Poirier et al. (2018) concluded that the best model included only lean body mass as a predictor of cooling rate (r^2^ = 0.14), although acknowledging the considerable residual error associated with this outcome. Regardless, given the high level of collinearity expected between variables entered into the stepwise regression and the known issues with this procedure (Harrell, 2015), plus the lack of sex as a fixed factor in these models, these conclusions are questionable. Recent evidence further nullifies the role of morphology in women’s unique cooling response, finding that the cooling rates of hyperthermic women with high or low body surface area-to-lean mass ratios were excellent (high 0.27°C∙min^−1^; low 0.26°C∙min^−1^) but comparable during CWI (10°C), and adiposity (r = 0.29, *p* = .28), lean body mass (r = −0.10, *p* = .70) and body surface area-to-lean mass ratio (r = 0.14, *p* = .97) was not correlated with cooling rates (Koenig et al., 2022).

All this considered, the responses of hyperthermic men and women to CWI cannot be definitively or solely attributed to morphological differences between the sexes with the evidence currently available and the attribute(s) that are responsible remain elusive. More data is required to conclusively identify the myriad of factors influencing sex specific and individual cooling responses during CWI. As the vast sum of data needed to realise this is likely unachievable with a human control trial approach, utilising medical data from field events with frequent EHS cases, as suggested by Pryor et al. (2022) may support the initial identification of characteristics that can be targeted in future randomized control trials.

One suggestion in an early review by Graham (1988) was a sex difference in metabolic sensitivity to heat loss. The review highlighted that at rest women cooled faster than men during CWI, however, the metabolic rate of the women was equivalent to the men (McArdle et al., 1984a). At rest, for the same degree of cooling women showed less of a metabolic response than men. When metabolic rate was fixed *via* exercise while immersed, the sex difference in cooling rate was absent (Hayward et al., 1975; McArdle et al., 1984b). In field studies involving divers, metabolic rate was self-selected and the responses of men and women resembled the resting data with female divers exhibiting a lower metabolic response to immersion and tolerating a 1°C greater decline in rectal temperature than the males (Kang et al., 1983; Shiraki et al., 1986). From this assessment the author proposed that women host a lower metabolic sensitivity to body cooling than men. Presumably, a lesser metabolic response contributes to greater cooling which may explain the lower metabolic heat production and rectal temperature values in the female compared to the male participants in Lemire et al.’s (2009) work.

Another theory suggests that the faster cooling rates in Lemire et al.’s (2009) female participants are the effect of a more prolonged elevation in the temperature of previously active and inactive skeletal muscles, which may increase the thermal conductivity of the limbs. In Kenny and Jay’s (2007) study, a greater decrease in mean arterial pressure caused by increased blood pooling in previously active limbs of women was said to explain higher limb temperatures. While this theory is physiologically plausible, it remains speculative as to the authors’ knowledge the effect of this physiological sex difference on heat transfer between the body and the environment has not been directly measured.

The smaller plasma volume relative to total body water in females (Retzlaff et al., 1969) may play a role in women’s response to CWI. As plasma volume dictates the blood flow available for peripheral heat exchange (Fortney et al., 1981), an association between blood volume and cooling rate is reasonable to suggest. Nevertheless, the expected smaller plasma volume seemingly contradicts logic in evidence of faster cooling rates during CWI (Lemire et al., 2009; Boehm and Miller, 2019). Likewise, mild dehydration (∼3%) *via* fluid restriction and exercise in the heat has been shown, though only marginally, to impair the cooling rate in a group of men and women during CWI (Butts et al., 2016). Whether this indicates the absence of plasma volume involvement in cooling in such scenarios or, speculatively, the actions of an unidentified compensatory responses in women to minimise the effect of their smaller blood volume, more data is required to explain the enhanced cooling rates observed.

Given the aforementioned absence of female-focused research on this topic, it is no surprise that female reproductive hormones’ effect on physiological responses to CWI post-hyperthermia has not been investigated. Female reproductive hormones (synthetic and natural) have distinct effects on many physiological systems, including thermoregulation (Baker et al., 2020; Giersch et al., 2020). As oestrogen and progesterone concentrations shift, so do core temperature and the onset threshold of sudomotor and vasomotor responses (Kolka and Stephenson, 1997). How this interaction translates to cold exposure is relatively understudied (Greenfield et al., 2021) though the local vasodilator effects of oestradiol may modulate women’s cooling capacity (Charkoudian and Johnson, 1999), and different hormonal profiles may alter shivering responses (Gonzalez and Blanchard, 1998). A recent review on sex differences in EHS risk reinforced this, highlighting that further research on the effect of oestradiol on cooling efficiency following heat stress is required (Giersch et al., 2022). Hence, the more rapid cooling response observed in females might be attributed partly to reproductive hormones that are known to have elaborate and multifaceted effects on women’s body temperature regulation.

## Discussion

More female data is needed to identify how women respond to CWI following exercise and heat-induced hyperthermia and understand the mechanisms driving their response. Given that in the available data, predominantly efficient cooling rates (> 0.15°C∙min^−1^) have been reported in women (Table 1; Armstrong et al., 1996; DeMartini et al., 2015; Koenig et al., 2022; Lemire et al., 2009), it is not believed that CWI is an ineffective cooling strategy for this population. Nevertheless, it remains questionable whether the current guidelines are optimised to protect women or could, in some circumstances, be harmful.

### Overcooling during cold water immersion

The risks associated with hyperthermia far outweigh those with overcooling, though many investigators advocate against overcooling hyperthermic individuals (Gagnon et al., 2010; Roberts et al., 2021). This is likely as the more core temperature falls below normal values, the greater the risk of blunting thermoregulatory function (Casa et al., 2007). If deep body temperature declines unabated, signs and symptoms of an impaired cardiovascular and neural function of increasing severity will present (e.g., disorientation, cardiac arrhythmias, loss of consciousness, ventricular fibrillation) (Bierens et al., 2016; Makranz et al., 2011). In severe circumstances, circulatory collapse or myocardial infarction may occur (Moran et al., 2003). Per recommendations, if the rectal temperature cannot be obtained on-site, the patient should be immersed in <10°C water for approximately 10–15 min (Casa et al., 2007, 2015). This immersion period assumes that an adequate cooling rate (>0.15°C∙min^−1^) is produced by the treatment and accounts for a range of pre-immersion core temperatures (40°C–∼42°C). Considering that women may cool faster than men and thus require a shorter immersion time to lower core temperature, 10–15 min may provide excessive cooling that drives body temperatures towards mild or severe hypothermia, especially in colder water. For instance, using the mean cooling rate in women reported by Koenig and colleagues (2022), 0.27°C∙min^−1^, the immersion time for an individual with a pre-immersion rectal temperature of 40°C, cooled to 38.6°C would equate to just 5.2 min, far less than the recommended immersion period of 10–15 min. For a core temperature beginning at 43°C, immersion time would equal 16.3 min, which is more in line with the recommendations. Notably, this cooling rate was achieved in 10°C water; thus, an even greater rate and shorter immersion time may have been observed in colder water. Despite its role in EHS diagnosis and treatment, the use and availability of rectal temperature assessment tools in the field is limited, even for health professionals like athletic trainers and emergency medicine providers (Mazerolle et al., 2010; Hirschhorn et al., 2021). Accordingly, it is critical that this time-duration guideline, which is most pertinent in real-world scenarios, is confirmed to be safe and effective for women.

### Post-immersion hypothermic afterdrop

A faster cooling rate may also be linked to a greater afterdrop in core temperature following immersion (Proulx et al., 2006). In a comparison of different water temperatures, the cooling rate of the male and female participants was significantly faster and a more substantial core temperature afterdrop was noted in ice water (2°C) compared to cold (14°C) and temperate (20°C) water conditions (Proulx et al., 2006). Anecdotal reports have noted that in more severe EHS cases (rectal temperature >42.5°C), individuals have demonstrated a hypothermic freefall post-immersion where rectal temperature values fell as low as 33.5°C despite ceasing immersion at a rectal temperature of 38.8°C (Gagnon et al., 2010). Though not reported, the cooling rates in these observed cases were likely optimized by the large thermal gradient created between the body and cold water and this may have contributed to the marked afterdrop reported.

If core temperature cooling rate and afterdrop are linked, and women are found to cool faster than men, a more severe afterdrop following CWI may occur in women. Additionally, if the recommended treatment protocol is followed, and thus the cooling rate is further optimised, the outcomes may be more severe. Attenuating the core temperature afterdrop is the primary purpose of the safety cooling limit which was developed from studies that involved participants of both sexes (Table 1; Proulx et al., 2006; Gagnon et al., 2010; 2012). Though, the above raises the question of whether the current limit for cold water (<10°C) of 38.6°C would be appropriate for females if they were found to produce a lower core temperature nadir following immersion.

## Conclusion

There is a paucity of data examining women’s response to CWI in treating EHS. Thus unsurprisingly, the current EHS guidelines are predominantly informed by research on males and have been accepted as appropriate for women without validation. Regardless, CWI is still an appropriate treatment for women with EHS, as the limited data indicates that women respond better than men to this treatment. A rapid cooling response, though seemingly beneficial for EHS treatment, could place women at risk of being overcooled if their core temperature is not closely monitored during and following immersion. The mechanism driving the rapid cooling has yet to be elucidated, with the logical explanation associated with the anthropometric differences between the sexes (known to alter other thermoregulatory properties) not appearing to be responsible. Future research is needed to 1) confirm that hyperthermic women cool more rapidly during CWI than hyperthermic men, 2) understand the mechanisms driving women’s cooling response, and 3) assess the suitability of the current EHS guidelines for women.
