# Supplementary material for: Treating exertional heat stroke: Limited understanding of the female response to cold water immersion
Source: Front Physiol. 2022 Nov 25;13:1055810. doi: 10.3389/fphys.2022.1055810 (PMC9732943; doi:10.3389/fphys.2022.1055810)
Supplement: Supplementary file 1 [file DataSheet1.pdf]

Treating Exertional Heat Stroke: Limited Understanding of the Female Response to Cold Water Immersion.

Author Affiliations

*Kate P Hutchins, k.hutchins@qut.edu.au, School of Exercise and Nutrition Sciences, Queensland University of Technology; Address: Kelvin Grove, QUT, Queensland 4059, Australia

Geoffrey M Minett, geoffrey.minett@qut.edu.au, School of Exercise and Nutrition Sciences, Queensland University of Technology; Address: Kelvin Grove, QUT, Queensland 4059, Australia

Ian B Stewart, i.stewart@qut.edu.au, School of Exercise and Nutrition Sciences, Queensland University of Technology; Address: Kelvin Grove, QUT, Queensland 4059, Australia

*Corresponding Author

Code and data available at https://github.com/katehutchins/female-CWI.

Secondary Analysis Method

Absolute body surface area, surface area to lean body mass ratio and rectal temperature cooling rates were attained from Figure 4A and 4B in Lemire et al. (2009) using WebPlotDigitizer. The mean and standard deviation (SD) of this data was confirmed to align with the mean and SD reported for males and females in Table 1 and Table 3 from Lemire et al. (2009). Analysis was completed using R (v 4.1.2; R Core Team 2021). The values from correlation tables between cooling rate, absolute surface area, lean body mass and surface area to lean body mass ratio were inspected for highly correlation variables for the whole data set (n = 19), and a female only (n = 9) and male only (n = 10) subset of the data. Pearson product moment correlation coefficients were calculated to determine if surface area to lean body mass ratio or lean body mass was correlated with rectal temperature cooling rates. Correlations were completed for the whole data set and for the male and female data subsets. Confidence intervals (95%) were verified via Bootstrapping analysis using 1000 replications and the BCa method. Linear regression of whole data set and male and female subsets were used to assess the relationship between cooling rate (dependent) and surface area to lean body mass ratio (independent variables) or cooling rate and lean body mass, sex and absolute surface area. The level of significance was set at $p < 0.05$ for all statistical comparisons.

Reference List

Lemire, B. B., Gagnon, D., Jay, O., & Kenny, G. P. (2009). Differences between sexes in rectal cooling rates after exercise-induced hyperthermia. *Med Sci Sports Exerc, 41*(8), 1633-1639. https://doi.org/10.1249/MSS.0b013e31819e010c

R Core Team (2021) R: a language and environment for statistical computing. Version 4.1.2. R Foundation for Statistical Computing, Vienna, Austria. https://www.R-project.org
